# Acid-sensing ion channels 1a (ASIC1a) inhibit neuromuscular transmission in female mice

**DOI:** 10.1152/ajpcell.00301.2013

**Published:** 2013-12-11

**Authors:** Francisco J. Urbano, Noelia G. Lino, Carlota M. F. González-Inchauspe, Laura E. González, Natalia Colettis, Lucas G. Vattino, Amanda M. Wunsch, John A. Wemmie, Osvaldo D. Uchitel

**Affiliations:** ^1^Laboratorio de Fisiología y Biología Molecular (LFBM), Instituto de Fisiología, Biología Molecular y Neurociencias (IFIBYNE, UBA-CONICET), Intendente Güiraldes 2160, Ciudad Universitaria, Buenos Aires, Argentina; and; ^2^Department of Psychiatry, Roy J. and Lucille A. Carver College of Medicine, The University of Iowa and Department of Veterans Affairs Medical Center, Iowa City, Iowa

**Keywords:** wire hanging, acid-sensing ion channels, motor nerve transmission, presynaptic modulation, extracellular acidosis

## Abstract

Acid-sensing ion channels (ASIC) open in response to extracellular acidosis. ASIC1a, a particular subtype of these channels, has been described to have a postsynaptic distribution in the brain, being involved not only in ischemia and epilepsy, but also in fear and psychiatric pathologies. High-frequency stimulation of skeletal motor nerve terminals (MNTs) can induce presynaptic pH changes in combination with an acidification of the synaptic cleft, known to contribute to muscle fatigue. Here, we studied the role of ASIC1a channels on neuromuscular transmission. We combined a behavioral wire hanging test with electrophysiology, pharmacological, and immunofluorescence techniques to compare wild-type and ASIC1a lacking mice (ASIC1a ^−/−^ knockout). Our results showed that *1*) ASIC1a ^−/−^ female mice were weaker than wild type, presenting shorter times during the wire hanging test; *2*) *s*pontaneous neurotransmitter release was reduced by ASIC1a activation, suggesting a presynaptic location of these channels at individual MNTs; *3*) ASIC1a-mediated effects were emulated by extracellular local application of acid saline solutions (pH = 6.0; HEPES/MES-based solution); and *4*) immunofluorescence techniques revealed the presence of ASIC1a antigens on MNTs. These results suggest that ASIC1a channels might be involved in controlling neuromuscular transmission, muscle contraction and fatigue in female mice.

acid-sensing ion channels (ASIC) open in response to extracellular acidosis (47). ASIC are members of the degenerin/epithelial Na^+^ channel protein family, initially described to be blocked by amiloride (29). Four genes generate at least six different subunits after alternative splicing: ASIC1a, -1b, -2a, -2b, -3, and -4. Homomeric or heteromeric tetramers can form functional channels. ASIC pH sensitivity and Ca^2+^ permeability depend on their subunit stoichiometry (5, 7, 8, 12, 21, 52). Specific pharmacological tools are available to block ASIC channels (18, 19), some of which have been tested as analgesics (47, 51). Psalmotoxin-1 is widely used to determine the physiological role of homomeric ASIC1a channels (19). ASIC1a channels have been involved in ischemia and epilepsy (22, 50, 52, 56). In addition, they play a role in fear circuitry and other psychiatric pathologies (13, 47).

Using in situ hybridization and immunochemistry techniques, ASIC1a, ASIC-2a, and ASIC-2b subunits have been found in brain tissue, mainly located at postsynaptic terminals (3, 11, 19, 35, 45, 46). ASIC have been suggested to be located on presynaptic terminals because of their ability to inhibit K^+^-Ca^2+^-dependent channels (41) and their effects on frequency of excitatory minis (12).

Transient acidification of the synaptic cleft takes place by the corelease of H^+^ and neurotransmitter after fusion of synaptic vesicles with the presynaptic membrane (47). Accordingly, the presence of presynaptic ASIC has been suggested to be a physiological “break” of neurotransmitter release-mediated acidification-basification loops (47). However, direct evidence of presynaptic ASIC is lacking.

In MNTs from skeletal muscles, a high vesicular proton concentration has been associated with the transient acidification of the synaptic cleft that would take place during ACh (acetylcholine) release (pH ∼ 5.5; 4, 39). Protons have been described to interact with multiple extracellular sites of nicotinic ACh receptors, decreasing the effective EC_50_ of the agonist and accelerating gating kinetics (1). Transgenically expressed pH-sensitive, yellow fluorescent protein in MNT from mouse skeletal muscle levator auris longus allowed Zhang et al. (55) to demonstrate that intracellular acidification (pH range 6.7–6.9, for ∼10 s) occurs during the initial phase of high-frequency stimulation (55). Afterward, a long-lasting intracellular alkalinization was observed in presynaptic MNTs, mediated by the massive incorporation of vesicular H^+^-ATPase into plasma membrane (55). Such alternation in intracellular pH levels has been associated with a massive extrusion of H^+^ to the synaptic cleft that can last for a few seconds. In addition, during less physiological (2), too intense activity at high frequency, MNTs activity can induce a pH drop in skeletal muscle environment to values as low as 6.5 (27), contributing to muscle fatigue (20). Although such decrease in pH might activate ASIC (46, 47), little is known about the presynaptic role of ASIC1a during skeletal muscle contraction and fatigue.

Here we tested the role of ASIC1a channels on skeletal motor nerve function and synaptic transmission. We conducted behavioral wire-hanging experiments, comparing wild-type and ASIC1a-lacking mice (ASIC1a ^−/−^ knockout). We then combined electrophysiology and pharmacological tools to study skeletal motor nerve transmission. Finally, immunofluorescence techniques were used to reveal the presence of ASIC antigens on MNTs. Our results showed that female ASIC1a ^−/−^ lasted less time during hanging tests than female wild-type mice. Nonsignificant differences in hanging times were observed using male mice. These results suggested that female mice lacking ASIC1a channels were weaker than female wild type, most likely due to a higher degree of skeletal muscle fatigue. At the MNT level from female mice, our results suggest that ASIC1a were located presynaptically, inhibiting both spontaneous and nerve-evoked neurotransmitter release. ASIC1a were activated by extracellular local application of acid saline solutions (pH = 6.0; HEPES/MES-based solution) as well as during nerve stimulation (at both low and high frequencies). These results clearly associate ASIC1a channels to a presynaptic fine tuning of skeletal muscle contraction and fatigue during high-frequency stimulation.

## MATERIALS AND METHODS

### 

#### Animal protocols.

Animal protocols for handling mice were submitted in accordance with guidelines of the Animal Care and Use Committee and approved by the Animal Care Facility of the Marine Biological Laboratory (Woods Hole, MA) and by the University of Buenos Aires, Faculty of Exact and Natural Sciences.

#### Genotyping protocol to identify ASIC1a^−/−^ mice.

DNA samples were obtained from tail samples and standard PCR techniques were used. We used the following oligonucleotides in our reactions: ASIC-53: 5′-CCGCCTTGAGCGGCAGGTTTAAAGG-3′; ASIC-18: 5′-CATGTCACCAAGCTCGACGAGGTG-3′; and ASIC-NEO: 5′-TGGATGTGGAATGTGTGCGA-3′. ASIC-53 and ASIC18 were used to identify the wild-type allele, while ASIC-53 and ASIC-NEO were used to assess the ASIC1a ^−/−^ allele.

#### Wire-hanging test.

The wire-hanging test evaluates neuromuscular grip strength and motor function of wild-type and ASIC1a ^−/−^ mice of either sex, as previously described (14, 54). The test begins with the mouse hanging by its forelimbs in the middle of an elevated wire placed on top of a metallic container; the latency to fall is recorded. Mice that fell in <10 s were tested for a second time. The three first trials (T1–T3) were separated 24 h, while T4 and T5 were performed 72 h apart. T4 and T5 trials were performed to test whether differences in wire hanging times between wild-type and ASIC1a ^−/−^ mice could still be observed beyond mice learning, expected to occur during 24-h periods. During each trial, mice were tested three times per session (10-s interval). Maximum time recorded per session was 600 s. The average performance for each session is presented as the average of all trials. Testing typically lasted up to 2 wk. Mean times were normalized by weight for each mouse using the following equation: mean hang time (s) × [individual mouse weight (g)/mean weight (g)]. Male mean weights were 30.5 ± 1.5 g (wild type; *n* = 6) and 26.9 ± 1.6 g (ASIC1a ^−/−^; *n* = 8). Female mean weights were 22.9 ± 0.8 g (wild type; *n* = 6) and 25.1 ± 0.6 g (ASIC1a ^−/−^; *n* = 8).

#### Muscle dissection.

Wild-type mice (C57BL/6, either sex, 30–50 days old) were purchased from Jackson Laboratory. Either sex mice lacking ASIC1a were used to compare neuromuscular transmission with wild-type and wild-type + psalmotoxin-1 (10 nM) experiments.

Mice (either sex) were anesthetized with isoflurane (AErrane, Baxter Health Care, Mundelein, IL) and immediately exsanguinated. Levator auris longus muscles were excised and dissected in a low-buffer capability (i.e., 0.8 mM HEPES) saline solution containing 145 mM NaCl, 2.5 mM KCl, 2 mM CaCl_2_, 1 mM MgCl_2_, 0.8 mM HEPES, 30 mM glucose, pH 7.4, 290–300 mosM, continuously bubbled with O_2_. Glucose concentration was reduced to compensate osmolarity of either 10 mM HEPES- or 10 mM MES-modified saline solutions (17, 44).

In a few experiments, the bicarbonate-based artificial cerebrospinal saline solution (137 mM NaCl, 2.5 mM KCl, 2 mM CaCl_2_, 1 mM MgSO_4_, 12 mM NaHCO_3_, 1 mM Na_2_HPO_4_, and 11 mM glucose, pH = 7.4, equilibrated with a mixture of 95% O_2_-5% CO_2_) was used to dissect and study neuromuscular transmission from both wild-type and ASIC1a ^−/−^ mice. All experiments were performed at room temperature.

#### Electrophysiology.

Evoked (EPP) and spontaneous (MEPP) end-plate potentials were recorded with conventional intracellular microelectrodes filled with 3 M KCl (20–40 MΩ). Levator auris longus skeletal nerve-muscle preparations from mice were continuously bathed with fresh 0.8 mM HEPES-saline solution containing μ-conotoxin GIIIB (10 μM) to prevent muscle contraction.

After muscle impalement, nerve was stimulated with two platinum electrodes coupled to a stimulus isolation unit (Isolator-11, Axon Instruments). Neurotransmitter release facilitation was elicited by supramaximal nerve stimuli.

Quantal content was determined using the direct method (17, 44) during low (0.5 Hz)- and high (75 Hz)-frequency stimulation. The effect of EPP nonlinear summation was corrected using the formula QC = E/*m*[(1 − E)/V_m_], where E is the amplitude of an individual EPP, *m* is the mean amplitude of MEPP, and V_m_ is membrane potential. Amplitudes for both EPP and MEPP were normalized to V_m_ = −70 mV, assuming 0 mV as the reversal potential for ACh nicotinic receptors.

Paired-pulse ratio values (2nd EPP amplitudes/1st EPP amplitudes) were obtained during 20 Hz paired-pulse stimulation.

Intracellular recordings were obtained using an Axoclamp 2A amplifier in combination with a Digidata 1440A interface and commanded with pClamp 8.2 software (Axon Instruments, Molecular Devices, CA).

#### Confocal muscle immunofluorescence analysis and images processing.

Dissected whole levator auris longus skeletal muscles were fixed in 1% paraformaldehyde overnight at 4°C, permeabilized with 1% Triton X-100 for 10 min, and incubated with the primary antibodies diluted in PBS containing 3% BSA, 0.1 M l-lysine, and 0.075% Triton X-100 for 16–20 h at 4°C. Rabbit anti-ASIC1a polyclonal antibody was used in a dilution of 1:50, while mouse anti-synaptophysin antibody was used in a 1:200 dilution. Binding of the primary antibody was revealed with a goat anti-mouse IgG attached to rhodamine (1:64) and a goat anti-rabbit IgG coupled to Alexa 488 (1:1,000). Rhodamine-conjugated α-bungarotoxin (1:2,000) was used as a postsynaptic MNT marker, due to its high affinity for acetylcholine receptors. After extensive washing with PBS, muscles were mounted in PBS-glycerol (1:1), covered with coverslips, and sealed with nail polish.

Images were obtained using a FV300 confocal fluorescence microscope (Olympus Optical, Tokyo, Japan) equipped with an image-acquisition system and Fluoview 3.3 software (Olympus Optical). Sequential scanning of slices with argon (λ: 488 nm) and helium-neon (λ: 543 nm) lasers was performed to reduce bleeding-through of fluorescence signal. Laser intensity as well as voltage, gain, and offset configuration of each photomultiplier were kept constant for every experiment. An Olympus 60× oil-immersion PlanApo objective (NA: 1.4) was employed for analysis of coverslip-mounted muscles. Stacks of 20 confocal planes (1-μm interval) were acquired for each channel and subsequently analyzed with ImageJ 1.42 software (National Institutes of Health, Bethesda, MD).

Immunofluorescence colocalization analysis was performed by Manders′ coefficient measurement using JACoP plug-in from ImageJ 1.42 software. This coefficient is an ideal choice when analyzing images with very different channel intensities (9). Setting the threshold to the estimated value of background helped to overcome its high sensitivity to noise.

Manders′ coefficient for channel A (ASIC1a immunofluorescence, green) was calculated as follows:

$$M=\frac{\sum_{1}A_{i,coloc}}{\sum_{1}A_{i}}$$

with *A*_*i*_ and *B*_*i*_ being *channel A* and *B* gray values, respectively, and *A*_*i, coloc*_ being *A*_*i*_ if *B*_*i*_ > background, or 0 if *B*_*i*_ = background. To strengthen our calculations about localization of ASIC1a channels in MNTs, Pearson's correlation coefficients (9) were also calculated for the same set of images used for Manders' coefficients calculations.

#### Toxin and chemicals.

Alpha-bungarotoxin and all salts of analytical grade were purchased from Sigma (St. Louis, MO), and μ-conotoxin GIIIB and psalmotoxin-1 were purchased from Alomone Labs (Jerusamen, Israel). Sucrose was purchased from Merck (Darmstadt, Germany).

#### Data presentation and statistics.

MEPP were analyzed using Mini Analysis software (Version 6.0.7; Synaptosoft, Chapel Hill, NC; www.synaptosoft.com). The threshold for minidetection was set at threefold the RMS baseline noise. Average data are expressed and plotted as means ± standard error of the mean (SE). Statistical significance was determined by using Student's *t*-test and median tests for unmatched pairs (*P* < 0.05) or one-way ANOVA (*P* < 0.05).

## RESULTS

### 

#### Wire hanging times were significantly shorter for female ASIC1a ^−/−^ compared with wild-type mice.

**Fig. 1.**
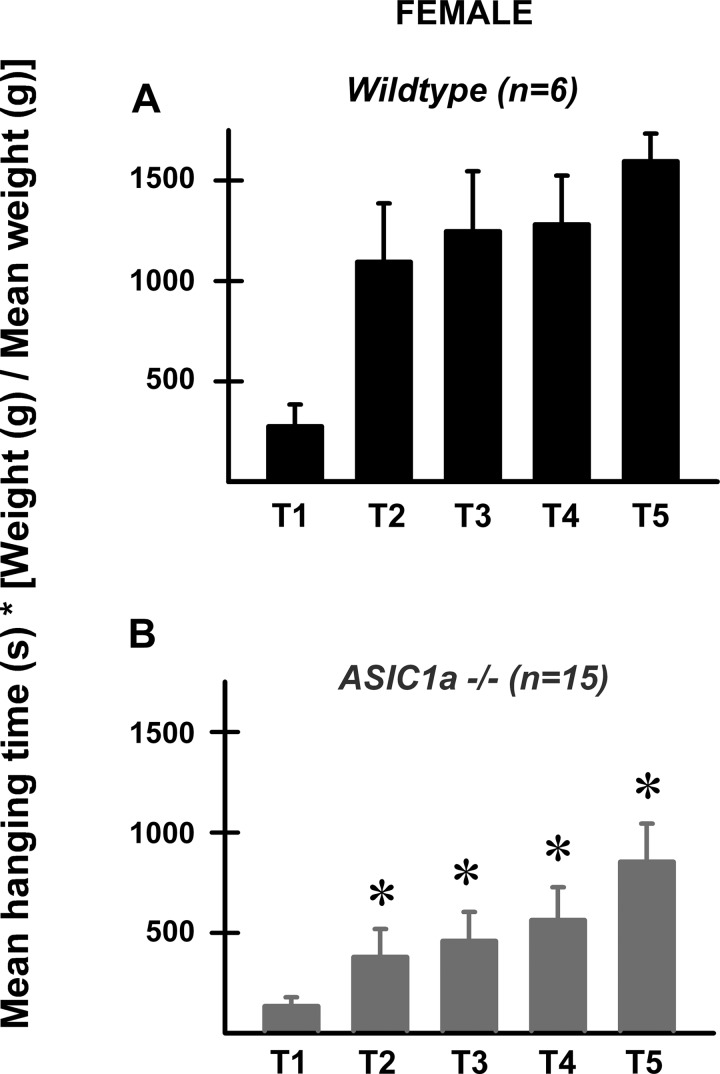
Acid-sensing ion channel 1a (ASIC1a) channels differently influenced hanging times in female mice. We used wire hanging test to evaluate neuromuscular grip strength and motor function of female wild-type and ASIC1a ^−/−^ mice. The three first trials (T1–T3) were separated by 24 h, while T4 and T5 were performed 72 h apart. Mean times were normalized by weight for each mouse using the following equation: mean hanging time (s) × [individual mouse weight (g)/mean weight (g)]. Wild-type (WT) female mice (*A*; black bars) lasted significantly more time (T2 through T5) compared with ASIC1a ^−/−^ female mice (*B*; gray bars). *P < 0.05 comparing wild-type and ASIC1a ^−/−^ times (Bonferroni post hoc test after 2-way ANOVA).

Wire hanging test was performed for ASIC1a ^−/−^ knockout and wild-type mice. Five trials of experiments were performed; four of them separated by 24 h except for the fifth trial which was separated 72 h apart from the fourth one. Average hanging times normalized according to the animals' weights are shown in Fig. 1. Wire hanging times were significantly longer for female wild-type (Fig. 1*A*; *n* = 6 mice) compared with ASIC1a ^−/−^ (Fig. 1*B*; *n* = 15 mice) after the second trial (*P* < 0.05, Bonferroni post hoc test after two-way ANOVA). In contrast, wild-type and ASIC1a ^−/−^ male did not differ in their hanging times (*n* = 6 and 8 mice, respectively; data not shown). However, wire hanging times were significantly smaller for male wild-type compared with female wild-type mice from T2 to T5 (male wild-type hang times were: T2 = 162 ± 120 s; T3= 315 ± 127 s; T4 = 472 ± 297 s, and T5 = 949 ± 337 s; *n* = 5; Student's *t*-test, *P* < 0.05 compared with wild-type female hang times shown on Fig. 1*A*).

These results suggest that at least in females ASIC1a channels are involved in controlling skeletal muscle fatigue.

#### ASIC1a channels presynaptically control transmitter release at MNTs.

We studied synaptic transmission using MNTs from levator auris longus skeletal muscle. Miniature end-plate potential (MEPP) frequencies, a presynaptically dependent parameter of MNTs, were measured by intracellular recordings of levator auris longus muscle fibers submerged in a 0.8 mM HEPES (2 mM [Ca^2+^]/1 mM [Mg^2+^]) saline solution. MEPP frequencies from both males and female mice were recorded from wild-type, wild-type + psalmotoxin-1 (10 nM) and ASIC1a ^−/−^ MNTs.

**Fig. 2.**
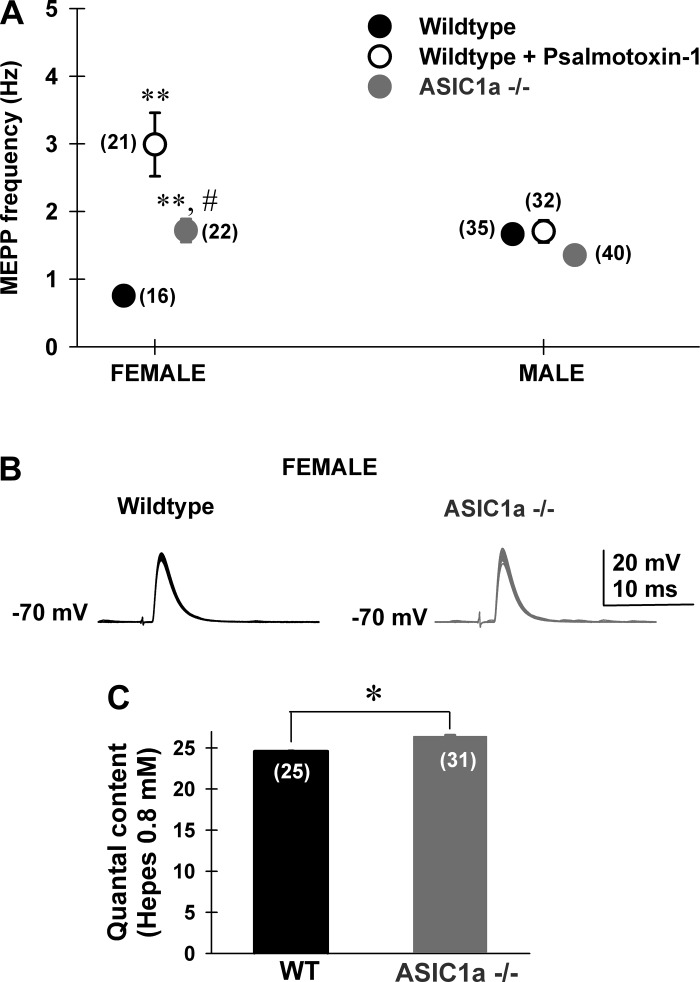
ASIC1a channels presynaptically control transmitter release in motor nerve terminals from female mice. *A*: average spontaneous miniature end-plate potential (MEPP) frequencies obtained from female (*left*) and male (*right*) wild-type (filled black circles), wild type + psalmotoxin-1 (10 nM, open black circles), and ASIC1a ^−/−^ (filled gray circles) levator auris longus muscle motor nerve terminals (MNTs). In female mice, wild-type frequencies were significantly lower compared with both wild type + psalmotoxin-1 (10 nM) and ASIC1a ^−/−^ [1-way ANOVA, *F*(2,61) = 23.1; Bonferroni post hoc test, *P* < 0.001]. In turn, wild-type + psalmotoxin-1 (10 nM) frequencies were significantly higher than ASIC1a ^−/−^ (Bonferroni post hoc test, *P* = 0.01). In male mice, frequencies were not significantly different (Bonferroni post hoc test, *P* = 0.14). Numbers in parenthesis correspond to the number of MNTs studied at each condition (2–4 levator auris longus muscles/condition). **P < 0.001 compared with wild type; #*P* < 0.05 comparing wild type and ASIC1a ^−/−^. *B*: representative end-plate potentials (EPP) from female wild-type (black traces) and ASIC1a ^−/−^ MNTs (gray traces) during 0.5-Hz nerve stimulation (in the presence of 10 μM conotoxin GIIIB, to prevent muscle contraction) at a 0.8 mM HEPES and 2 mM [Ca^2+^]/1 mM [Mg^2+^] saline solution. *C*: mean quantal content determined by the direct method (see materials and methods) was significantly higher in ASIC-1a ^−/−^ female mice (gray bar, *n* = 31 MNTs) compared with wild-type female mice (black bar; *n* = 25 MNTs, *P < 0.05, Student's *t*-test).

In female mice, MEPP frequencies in ASIC1a ^−/−^ mice were significantly higher than in wild type (Bonferroni post hoc test, *P* = 0.01, Fig. 2*A*, *left*). Furthermore, in wild-type MNTs treated with psalmotoxin-1 (10 nM; *n* = 21 MNTs) MEPP frequencies were also higher compared with both control wild-type (*n* = 16 MNTs) and ASIC1a ^−/−^ (*n* = 22 MNTs) [one-way ANOVA, *F*(2,61) = 23.1; Bonferroni post hoc test, *P* < 0.001, Fig. 2*A, left*]. Male MNTs presented nonsignificant differences in MEPP frequencies between genotypes (wild type, *n* = 35 MNTs; ASIC1a ^−/−^, *n* = 40 MNTs) or psalmotoxin-1 (10 nM; *n* = 32 MNTs) treatment (Bonferroni post hoc test, *P* = 0.14, Fig. 2*A, right*). Thus we continued characterizing ASIC1a role on synaptic transmission using female mice. Quantal content was compared between female wild-type and ASIC1a ^−/−^ MNTs (0.8 mM HEPES-based saline solution containing 2 mM [Ca^2+^]/1 mM [Mg^2+^] + 10 μM μ conotoxin GIIIB), during low-frequency nerve stimulation (direct method, corrected for nonlinear summation, see materials and methods). Larger quantal content values were obtained from ASIC1a ^−/−^ MNTs, compared with wild type (Fig. 2*C*, mean quantal content, wild type: 24.6 ± 0.14, *n* = 25 MNTs; ASIC1a ^−/−^: 26.4 ± 0.15, *n* = 31 MNTs; Student's *t*-test, *P* = 0.03), thus suggesting an inhibitory role of ASIC1a ^−/−^ at presynaptic MNTs during low-frequency stimulation.

**Fig. 3.**
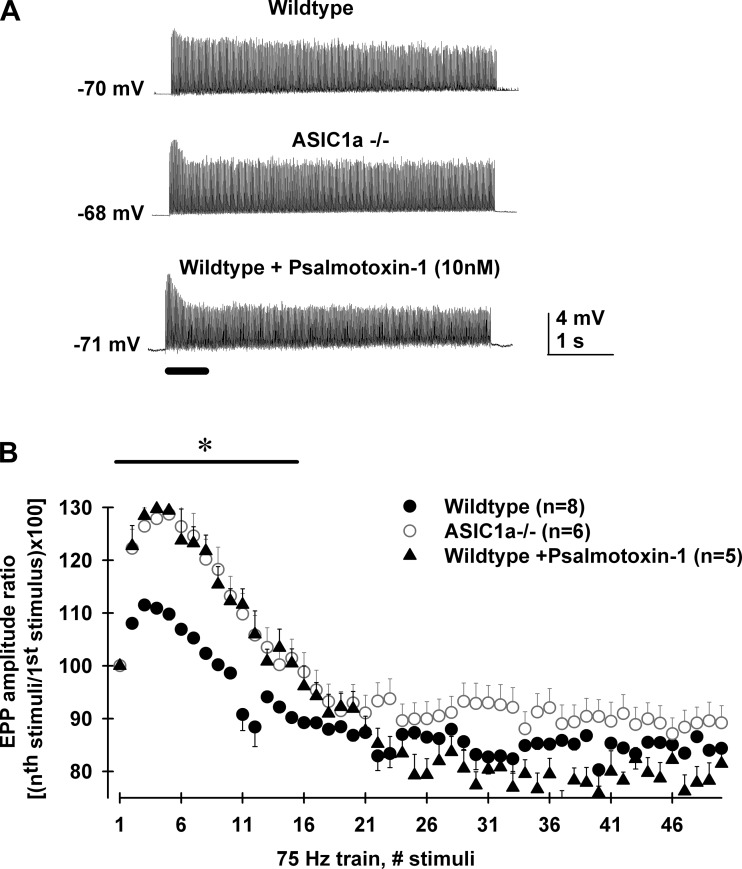
ASIC1a channels reduced neuromuscular transmission during 75 Hz/5 s stimulation train in motor nerve terminals of female mice. *A*: representative intracellular recordings of EPP are shown during nerve stimulation at 75 Hz (5 s long, 375 stimuli) using 0.8 mM HEPES/2 mM [Ca^2+^]/1 mM [Mg^2+^] + 10 μM μ-conotoxin GIIIB saline solution. EPP facilitate during the first stimuli and then undergo depression until a steady state was reached (top trace, wild type). Interestingly, EPP from ASIC1a ^−/−^ female mice (mid trace) and female wild type + psalmotoxin 1 (10 nM; bottom trace) presented greater initial facilitation compared with those from wild-type female mice (top trace). *B*: average EPP amplitude ratio [(*n*th stimuli amplitude/1st stimulus amplitude) × 100] of the initial 50 stimuli shown in *A* for levator auris longus MNTs from wild type (filled circles, *n* = 8 MNTs), ASIC1a ^−/−^ (open circles, *n* = 6 MNTs), and wild type + psalmotoxin-1 (10 nM; filled triangles, *n* = 5 MNTs). Wild-type ratios were significantly smaller than both wild-type + psalmotoxin-1 (10 nM) and ASIC1a ^−/−^ ones [one-way ANOVA, *F*(2,87) = 4.3; wild type vs. ASIC1a ^−/−^, wild type + psalmotoxin-1 (10 nM), Bonferroni post hoc test, *P* = 0.016]. Wild-type + psalmotoxin-1 (10 nM) and ASIC1a ^−/−^ ratios were not significantly different (Bonferroni post hoc test, *P* = 1.0). *P < 0.05 comparing wild-type with ASIC1a ^−/−^ and wild-type + psalmotoxin-1 ratios.

Long-lasting high-frequency stimulation showed that ASIC1a played an inhibitory role on neuromuscular transmission in female mice. During 75-Hz, 5-s-long nerve stimulation (0.8 mM HEPES saline solution containing 2 mM [Ca^2+^]/1 mM [Mg^2+^] + 10 μM μ-conotoxin GIIIB), EPP amplitudes from wild type MNTs augmented 15% during the initial 10 stimuli and then underwent depression until reaching a steady state (Fig. 3*A*). Interestingly, initial facilitation during 75 Hz trains was significantly higher in wild-type + psalmotoxin-1 (10 nM) and ASIC1a ^−/−^ compared with wild-type MNTs (Fig. 3*A*). Average EPP amplitude ratios, calculated as [(*n*th stimuli amplitude/1st stimulus amplitude) × 100] from wild type (*n* = 8 MNTs) were significantly smaller than those from wild type + psalmotoxin-1 (10 nM; *n* = 5 MNTs) and ASIC1a ^−/−^ [*n* = 5 MNTs; Fig. 3*B*, one-way ANOVA, *F*(2,87) = 4.3; wild type vs. ASIC1a ^−/−^, wild type + psalmotoxin-1, 10 nM; Bonferroni post hoc test, *P* = 0.016]. Moreover, wild type + psalmotoxin-1 (10 nM) and ASIC1a ^−/−^ ratios were not significantly different (Bonferroni post hoc test, *P* = 1.0).

Finally, we compared quantal content values using the first EPP of 75 Hz train. We only used one train per MNT to avoid the influence of synaptic plastic changes during repetitive train stimulation (i.e., accumulative depression). Quantal content values showed to be significantly different when comparing ASIC1a ^−/−^ with wild-type female MNTs (wild type, 10.4 ± 1.7, *n* = 8 MNTs; ASIC1a ^−/−^ 20.5 ± 3.6, *n* = 5 MNTs; Student's *t*-test, *P* < 0.01).

These results suggested that presynaptic ASIC1a channels are capable of inhibiting spontaneous and nerve-evoked neuromuscular transmission during low- and high-frequency stimulation in female mice.

#### ASIC1a-mediated presynaptic effects can be activated during local extracellular acidification.

We continued studying whether local transitory extracellular acidification might affect neuromuscular transmission in female wild-type levator auris longus skeletal muscles. If so, ASIC1a ^−/−^ MNTs should not present such modulation.

**Fig. 4.**
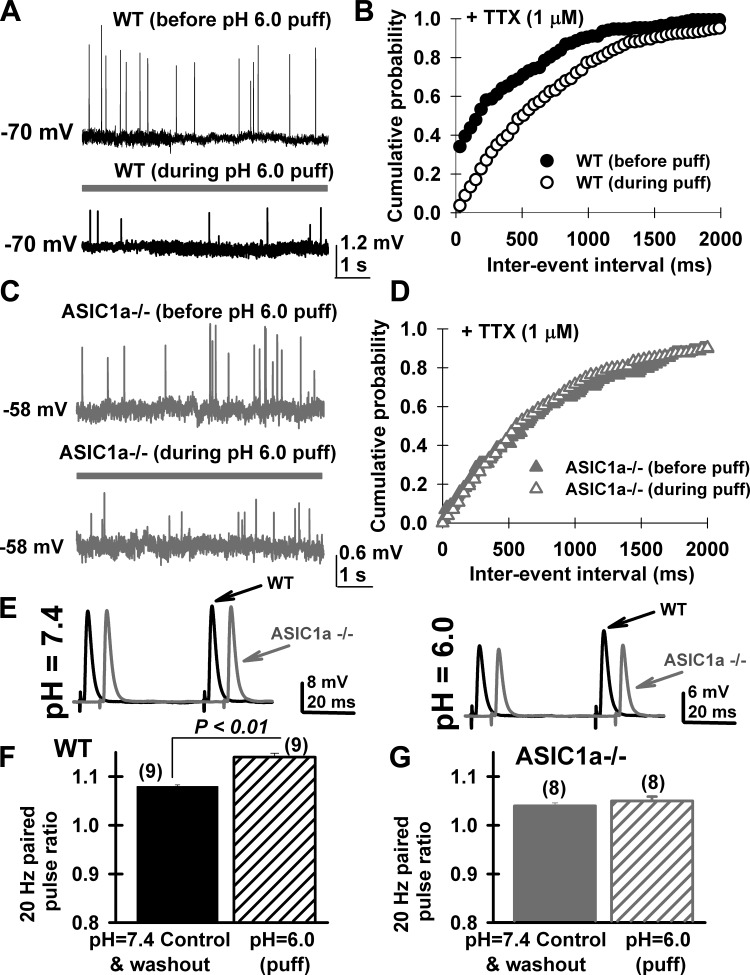
Extracellular acidification of MNTs with a pH = 6, HEPES/MES-based saline solution activated ASIC1a, affecting spontaneous miniature end-plate potentials (MEPP) and 20-Hz paired-pulse facilitation in MNTs from female mice. *A*: representative intracellular recordings of MEPP in the presence of TTX (1 μM) before (i.e., pH = 7.4, 10 mM HEPES saline solution) and during pressure application of an acid solution (i.e., pH 6.0, HEPES/MES-based modified solution, 6-s long application) to a MNT from a female wild-type levator auris longus muscle (black traces). *B*: cumulative interevent interval distribution graphs for the same neuromuscular junction shown in *A* before (black solid circles) and during pH = 6.0 application (black open circles). *C* and *D*: same as in *A*, but for a female ASIC1a ^−/−^ (gray traces and triangles) levator auris longus muscle. Superficial MNTs were optically selected from thin levator auris longus muscle fibers, and a “puff” pipette containing pH = 6.0, HEPES/MES-based saline solution was located at less than 100 μm from presynaptic terminals. *E*: representative 20-Hz paired-pulse traces from wild-type (black traces) and ASIC1a ^−/−^ (gray traces) female mice during local puff application using either pH = 7.4 (*left*) or pH = 6.0 (*right*) HEPES/MES-based saline solution. Each trace represents an average of eight individual traces. *F*: average paired-pulse ratio values from wild-type MNTs were significantly increased by local application of pH = 6.0, HEPES/MES-based saline solution (hatched black bar, Student's *t*-test, *P* < 0.007). *G*: paired-pulse ratio did not change when neuromuscular junction from ASIC1a ^−/−^ were bathed with a pH 6.0 solution, presenting similar mean paired-pulse ratios (hatched gray bar; Student's *t*-test, *P* = 0.3). Same results were observed in four different ASIC1a ^−/−^ MNTs. Numbers in parenthesis correspond to the number of MNTs studied at each condition (2 levator auris longus muscles/condition).

We recorded levator auris longus muscle fibers visually identified to have superficially accessible MNTs in a HEPES, 2 mM [Ca^2+^]/1 mM [Mg^2+^] saline solution. A puff pipette filled with a HEPES/MES-based saline solution at pH 6.0 was placed at less than 100 μm away from studied MNTs. A 6-s-long pressure application was locally delivered using a Picospritzer (Intracel). Local application of pH 6.0 MES-based solution reduced MEPP frequency (in 1 μM TTX) in wild-type MNTs (Fig. 4*A*, wild type), increasing interevent intervals (Fig. 4*B*). In contrast, no changes in MEPP frequency were observed when acid solution was applied onto ASIC1a ^−/−^ MNTs (Fig. 4, *C* and *D*). Local extracellular acidification reduced MEPP amplitudes at wild-type (mean MEPP amplitude at pH 7.4, 1.09 ± 0.08 mV; *n* = 7 MNTs; pH 6.0-mediated mean reduction, 28 ± 7%) and ASIC1a ^−/−^ MNTs (mean MEPP amplitude at pH 7.4, 0.90 ± 0.05 mV; *n* = 6 MNTs; Student's *t*-test, *P* > 0.05; pH 6.0-mediated mean reduction, 26 ± 10%) in a similar way (Student's *t*-test; *P* = 0.3). In all cases, acid-mediated effects were reversible after a 30-s washout with pH 7.4 solution. In addition, local acidification affected 20-Hz paired-pulse stimulation of wild type (*n* = 9 MNTs), but not ASIC1a ^−/−^ (*n* = 8 MNTs) (Fig. 4*E*). A transient increment of average ratios from 108 ± 0.3% to 114 ± 0.8% was observed during local acid application in wild-type MNTs (Fig. 4*F*; Student's *t*-test, *P* < 0.01). No changes in paired-pulse ratios were observed at ASIC1a ^−/−^ MNTs (Fig. 4*G*; 104 ± 0.5% to 105 ± 0.9% before and after acid puff, respectively; Student's *t*-test, *P* = 0.3). These results suggest that direct activation of presynaptic ASIC1a channels using local acidification is capable of inhibiting both spontaneous and nerve-evoked neuromuscular transmission.

#### Immunolabeling also shows ASIC1a channels to be located presynaptically.

We used immunofluorescence techniques to study the presence of ASIC antigens on neuromuscular junctions (MNTs) from mice levator auris longus muscles. Both pre- and postsynaptic markers were assayed in combination with polyclonal rabbit anti-ASIC1a antisera (Alexa 488, see materials and methods). Postsynaptic marker α-bungarotoxin (rhodamine-conjugated, α-BgTx-Rd) and presynaptic marker synaptophysin did not colocalize in MNTs from wild type (Manders' coefficient: 28 ± 3%; Pearson's coefficient: 36 ± 4%; *n* = 33 MNTs).

**Fig. 5.**
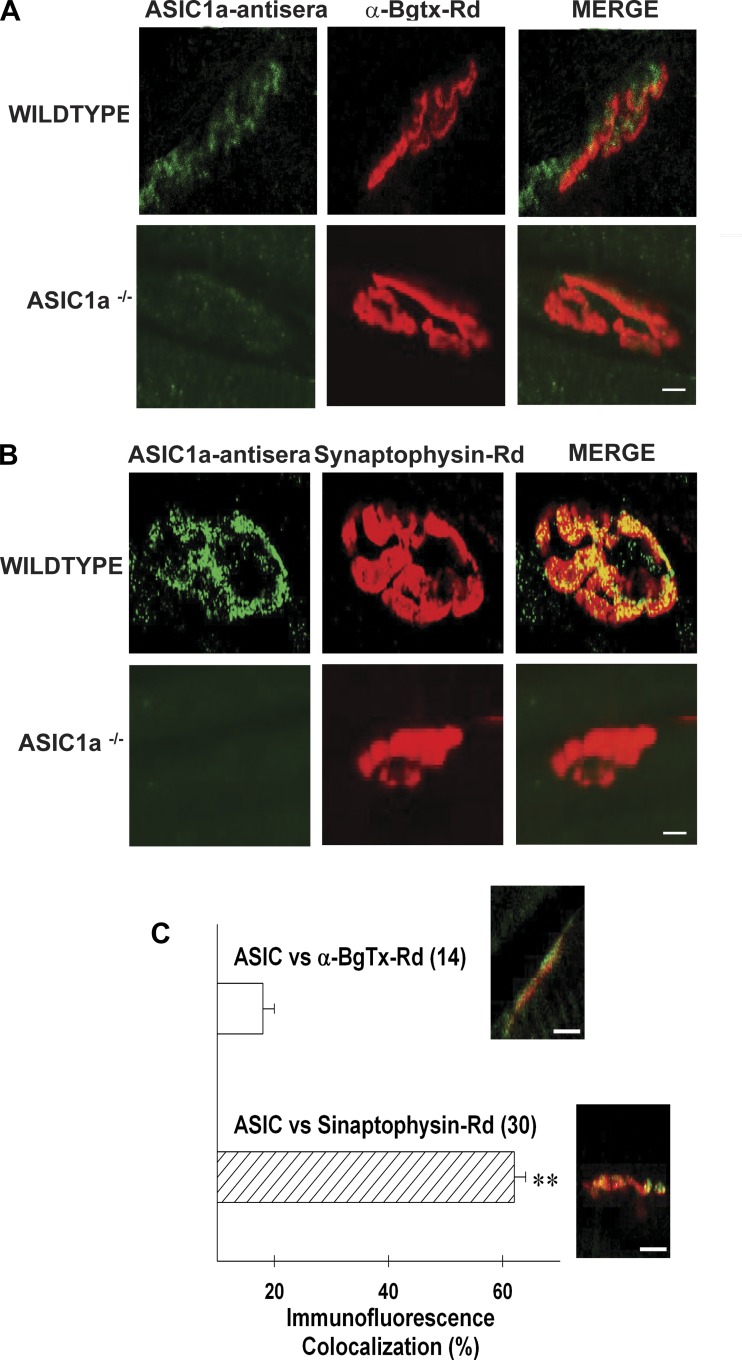
Immunofluorescence techniques showed ASIC1a channels to be located presynaptically in MNTs from female mice. *A*: double-labeled fluorescence confocal images using postsynaptic bounding marker rhodamine conjugated α-bungarotoxin (α-Bgtx-Rd; 1:2,000) and polyclonal rabbit anti-ASIC1a (1:50; secondary Ab anti-rabbit 1:1,000) immunostaining on a permeabilized levator auris longus muscle. *B*: double-labeled fluorescence confocal images using presynaptic anti-synaptophysin (primary Ab: mouse anti- synaptophysin 1:200; secondary Ab anti-mouse rhodamine-conjugated, 1:64) and polyclonal rabbit anti-ASIC1a antibodies (1:50; secondary Ab anti-rabbit Alexa 488-conjugated 1:1,000) on a permeabilized levator auris longus muscle. Scale bars = 10 μm. Confocal images in *A* and *B* represent results obtained in two different levator auris longus muscle preparations. Scale bars = 10 μm. *C*: bar graph comparing average Manders' coefficient for either ASIC1a and α-Bgtx-Rd colocalization (open bar) or ASIC1a and synaptophysin colocalization (hatched bar) using wild-type levator auris longus muscle preparations. Also, representative confocal images are shown as insets in each case. **P < 0.001 Student's *t*-test.

The postsynaptic marker α-bungarotoxin (rhodamine-conjugated, α-BgTx-Rd) immunostained MNTs from both wild-type and ASIC1a ^−/−^ mice. However, the rabbit anti-ASIC1a antisera signal was only detected in the wild-type mice (Fig. 5*A*). The presynaptic marker synaptophysin appeared to colocalize with ASIC1a antisera in wild-type mice (Fig. 5*B*). ASIC1a ^−/−^ signal was absent in MNTs identified by synaptophysin in the ASIC1a ^−/−^ MNTs (Fig. 5*B*). Average colocalization Manders' coefficient was significantly higher for presynaptic areas immunostained with synaptophysin (69 ± 5%; *n* = 30 MNTs) than for postsynaptic α-BgTx-Rd (25 ± 4%; *n* = 14 MNTs) (Fig. 5*C*, Student's *t*-test, *P* < 0.001). In addition, Pearson's coefficient also showed higher colocalization of synaptophysin with ASIC1a antisera in wild-type mice (50 ± 3%; *n* = 30 MNTs) compared with α-BgTx-Rd (30 ± 6%; *n* = 14 MNTs; Student's *t*-test, *P* < 0.001).

In summary, our results suggest that ASIC1a channels can modulate neuromuscular performance during wire hanging behavioral tests in females, and localized presynaptically at mouse MNTs. The absence of MNTs immunostaining by ASIC1-antisera on ASIC1a ^−/−^ muscles together with the great impact of psalmotoxin-1 (10 nM) on wild-type spontaneous and evoked transmitter release, suggest that ASIC1a channels located at mice MNTs are composed by homomeric ASIC1a subunits.

## DISCUSSION

From our results, the following findings can be stated. *1*) ASIC1a ^−/−^ female mice were weaker than female wild type, presenting shorter times during wire hanging tests. *2*) ASIC1a channels inhibited both spontaneous and nerve-evoked neurotransmitter release (using low and high frequency stimulation), suggesting the presynaptic location of these channels at individual MNTs-ACh releasing sites. *3*) ASIC1a-mediated effects were emulated by extracellular local application of acid saline solutions (pH = 6.0; HEPES/MES-based solution). *4*) Using indirect immunofluorescence we showed the presence of ASIC1a antigens on presynaptic MNTs.

Electrophysiological results presented here suggested an inhibitory role of ASIC1a channels. Indeed, the absence of ASIC1a channels from birth (i.e., ASIC1a ^−/−^ mice) resembled psalmotoxin-1 effects on neuromuscular transmission, suggesting no compensatory effect by other ASIC subunits. We took advantage of the thin muscle levator auris longus to locally apply acid solutions at pH 6.0 onto single MNTs (17), which was able to reduce spontaneous MEPP frequency while enhancing paired-pulse facilitation values. The latter effect suggested the activation of inhibitory ASIC1a during pH 6.0 local application, which would reduce the probability of release during the first stimulus, consequently enhancing EPP amplitudes during the second stimulus (i.e., increasing paired-pulse ratio values). However, contrary to our results an increment in MEPP frequency was described in the rat diaphragm following bath acidification (26). This apparent contradiction might be related to the speed of pH change, being in our case a rapid and limited puff application vs. a slow and muscle-wide perfusion used by Hubbard et al. (26). Indeed, longer time courses of acidification have been recently demonstrated to reduce the number of ASIC1a channels available to be open (32), which would turn an expected fast inactivation of transmitter release into facilitation when ASIC1a were rapidly passing from open to inactivated states. It is especially difficult to compare ASIC1a activation during both sets of experiments, since the duration of local acid puffs might change the recovery from inactivation of ASIC1a channels (32) while acidification of the cleft during high-frequency stimulation might be taking place in a totally different way (i.e., shorter but more robust acidifications might take place; see 12, 27, 55). It could be expected that presynaptic ASIC1a during the 6-s-long local acidification used in this work might activate during a few hundreds of milliseconds to then be inactivated.

Understanding how Ca^2+^-permeable ASIC1a (47, 52) can exert an inhibitory modulation of presynaptic transmitter release is specially challenging. Presynaptic effects of ASIC1a channels on the size of a readily releasable pool of synaptic vesicles or on its refilling have been ruled out in a previous work using hippocampal excitatory synapses from ASIC1a ^−/−^ (12), although they might be affecting endocytosis (55). One possible explanation might be related to the association of ASIC1a to Ca^2+^-dependent K^+^ channels, BK (41), that is known to inhibit those channels under physiological extracellular pH while releasing such inhibition during acidification. In this regard, experiments performed in our laboratory using charybdotoxin (50 nM; a potent BK and intermediate Ca^2+^-K^+^ channel blocker) neither affected EPP amplitudes during high-frequency 75-Hz stimulation nor prevented the reduction in MEPP frequency during local acidification (*n* > 3 MNTs; data not shown). Such apparent contradiction with a previous work (41) might be related to the fact that activation of BK is supposed to last for only few tens of milliseconds, but not for the long periods of local acidification used in this work, apart from the absence of a direct link between what was studied in HEK cells and synaptic transmission models like mouse MNTs. Other mechanisms such as the activation of intracellular pathways like Ca^2+^-dependent calmodulin kinase II (CaMKII) and others, or the activation of other subtypes of K^+^-channels may also take place. Those studies using MNTs still need to be done.

Sex differences in skeletal muscle fatigue (classically defined as a decrease in the maximum force that a muscle can generate after repetitive stimulations) have been previously reported in humans (24, 30). It is generally accepted that women are more resistant to skeletal muscle fatigue than males during sustained contractions of the elbow flexors (40), the hand-grip muscles (48), among others (25). However, other authors have described that female resistance to fatigue declines as skeletal muscle contractions increase (25). Such relationship between fatigue and muscle contraction intensity is particularly important because the existence of a presynaptic neuromuscular impairment during repetitive stimulations has been suggested to underlie fatigue-resistance differences between males and females (25). However, detailed studies about this possibility still need to be done. In this work, we took advantage of wire hanging tests, previously used to evaluate neuromuscular grip strength and motor function in mice (14, 54) to describe that female wild-type mice were also more resistant to fatigue than males. The fact that ASIC1a ^−/−^ female mice showed shorter times from T1 through T5 suggested long-lasting neuromuscular-dependent impairment rather than learning-related processes. Mechanisms underlying sex differences might vary with age (30). The impairment of neuromuscular activation after heavy resistance exercise has been mainly described in skeletal muscles from males (24). In this study, a wire hanging test showed that ASIC1a ^−/−^ female mice are less resistant to fall than males, and we also described that different ASIC1a-mediated spontaneous activity was present when male and female MNTs were used. Therefore, ASIC1a-mediated modulation of transmitter release might be one of the possible links behind sex differences in skeletal muscle performance during repetitive, high-intensity contractions. It is important to mention that the opposite differences have been described for muscle fatigue in ASIC3 ^−/−^ mice. Indeed, muscle fatigue increased in male but not female ASIC3 ^−/−^. Thus it can be hypothesized that both ASIC3 channels and testosterone are necessary to protect against muscle fatigue in males (10), although in this case ASIC3 channels might be acting through postsynaptic mechanisms in skeletal muscle tissue (23). Finally, less fatigue during incremental isometric exercise has been described in elder humans compared with young people (30). Further experiments are needed to characterize how the ASIC1a role in MNTs might change with age. In addition, ASIC3 receptors act in conjunction with transient receptor potential vanilloid type 1 (TRPV1) receptors activated by heat, acid, or endocannabinoids to detect metabolites that cause muscle pain and fatigue (33). However, in this study we provide a series of results that strongly suggest the presence of homomeric (i.e., 10 nM psalmotoxin-1-sensitive) presynaptic ASIC1a channels that would act as a “synaptic activity break” in females, inhibiting neurotransmitter release in mice MNTs from levator auris longus muscles.

At the synaptic level, previous studies have shown ASIC1 ^−/−^, but not ASIC2 ^−/−^, to have facilitatory effects on repetitive synaptic transmission (12). ASIC1a channels can associate with clathrin at presynaptic terminals while ASIC2a is associated with dendritic, postsynaptic MAP-1A (16) and PSD-95 (53). In agreement with these reports, here we showed that ASIC1a channels can control neuromuscular transmission in mice. In the present study, ASIC1a channels presented an inhibitory effect, in such a way that pharmacological (i.e., using 10 nM psalmotoxin-1) and genetic (i.e., using ASIC1a ^−/−^ mice) disruption of ASIC1a resulted in an enhancement of synaptic activity at MNTs.

Absence of immunofluorescent colocalization observed using ASIC1-antiserum in ASIC1a ^−/−^ mice (quantified using both Manders' and Pearson's coefficients) plus the similarity of results when comparing psalmotoxin-1 (10 nM) and ASIC1a ^−/−^ conditions on our presynaptically related observations (i.e., changes in MEPP frequency and enhanced facilitation during high-frequency stimulation) strongly suggest the presence of homomeric ASIC1a channels on presynaptic sites of levator auris longus MNTs from female mice.

**Fig. 6.**
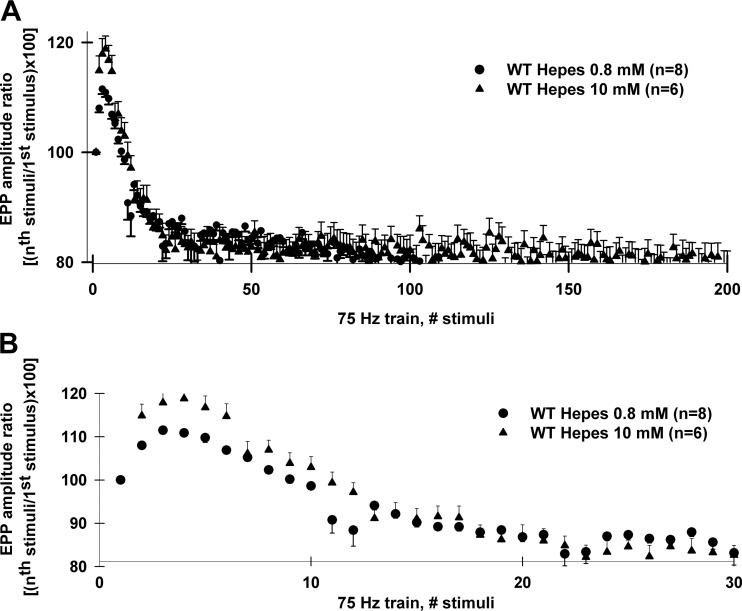
ASIC1a activation could be prevented by increasing the buffering capability of the extracellular saline solution in MNTs from female mice. *A*: during nerve stimulation at 75 Hz (5-s-long train) levator auris longus neuromuscular transmission from wild type presented bigger initial facilitation when a 10 mM HEPES saline solution was used (black triangles) compared with 0.8 mM HEPES solution (black circles). Both saline solutions contained 2 mM [Ca^2+^]/1 mM [Mg^2+^] + 10 μM μ-conotoxin GIIIB. EPP amplitude ratios were calculated as [(*n*th stimuli amplitude/1st stimulus amplitude) × 100]. 0.8 mM HEPES solution average ratios correspond to the same experiments showed in Fig. 5. *B*: detailed plot of the same average ratios shown in *A* corresponding to the initial 30 stimuli.

During repetitive synaptic activity, presynaptic motor nerve terminals from levator auris longus muscles are known to dynamically change its intracellular pH from an initial Ca^2+^-dependent acidification to a long-lasting alkalinization (55). Importantly, intracellular alkalinization is achieved after vesicular ATPase is incorporated into plasma membrane during massive vesicular fusion (55). Consequently, a massive extrusion of H^+^ to the synaptic cleft can be expected to last for a few seconds. This acidification window is sufficient to allow ASIC1a channels to open, having an impact on motor nerve transmitter release prior to desensitization (32). In our experiments, extracellular acidification was most likely to be exacerbated during the use of the low-buffer-capability saline solution composed by 0.8 mM HEPES. In agreement with this hypothesis, increasing HEPES concentration (up to 10 mM) could abolish the acidification of the neuromuscular cleft, thus preventing the activation of ASIC1a channels which would induce the neurotransmitter release facilitation observed during 75-Hz train stimulation (Fig. 6). Furthermore, when acid saline solutions were locally applied, ASIC1a-mediated effects were reproduced in wild type but not in ASIC1a ^−/−^ mice.

**Fig. 7.**
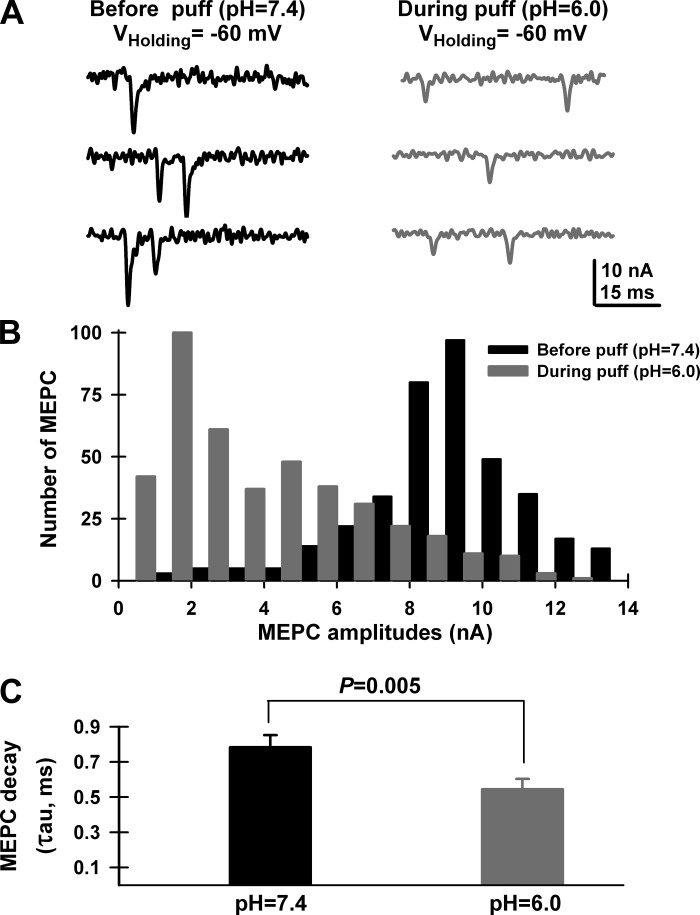
Postsynaptic nicotinic ACh receptors were blocked during local bathing of MNTs with a pH = 6.0, MES-based saline solution in MNTs from female mice. *A*: representative miniature end-plate currents (MEPC) recorded from a wild-type levator auris longus muscle fiber using the two-electrode voltage clamp technique (TEVC), with a 0.8 mM HEPES-based saline solution in the presence of TTX (1 μM). MEPC were recorded from the same muscle fiber at a pH = 7.4 (black lines) and during pressure application of an acid solution (blue lines, pH 6.0, MES-10 mM based modified solution, 6-s-long application). Note how MEPC amplitudes were drastically reduced in the presence of an acid saline solution (*B*), presenting shorter decay times than at pH 7.4 (*C*). *C*: MEPC decay time (τ) values were obtained by fitting decay times to single exponentials using Clampfit 10 software. A minimum of 30 MEPC were used for each condition. Student's *t*-test comparison of tau values showed that MEPC at pH = 6.0 had significantly shorter decay times compared with pH = 7.4 (Student's *t*-test; *P* = 0.005).

ASIC-mediated currents have been extensively described using extracellular local application of acid solutions in neuronal cultures (4, 6, 8, 12, 32, 35, 36). Although presynaptic MNTs cannot be accessed using patch electrodes, our experiments using locally applied “puffs” on visually selected superficial MNTs showed clear alterations at both spontaneous (MEPP) and nerve-evoked (20-Hz paired-pulse stimulation) neuromuscular transmission from wild-type but not ASIC1a ^−/−^ mice. Both parameters are classically known to be dependent on presynaptic mechanisms, directly suggesting the presence of presynaptic ASIC1a channels. The fact that at low-frequency stimulation, quantal content values were increased in the ASIC1a ^−/−^ mice could suggest the existence of a basal concentration of protons at the neuromuscular synaptic cleft resulting from the spontaneous release of synaptic vesicles. Such “autocrine” protons release was previously demonstrated in HEK293 cells (31). In addition, the clear reduction in MEPP amplitudes also suggested postsynaptic alterations during local acid application. Since postsynaptic effects of H^+^ have been associated to a speeding up of decay times of nicotinic acetylcholine receptors (1), we studied the effect of local acidification during miniature end-plate currents (MEPC) recorded using two-electrode voltage clamp (43). MEPC amplitudes were reduced in the presence of a pH = 6.0 acid saline solution and presented significantly faster decay times compared with physiological pH = 7.4 (Fig. 7). Therefore, the [H^+^]-dependent amplitude reduction observed during local acid application was related to nicotinic AChR conformational changes. Nevertheless, changes in postsynaptic input resistance related to the opening of ASIC-3 channels known to be expressed on muscle cells (23) could also be responsible for the observed reduction in MEPP amplitudes during local acidification.

Muscle fatigue is associated with a number of clinical diseases, including chronic pain conditions, such as chronic fatigue syndrome and fibromyalgia (15, 28, 37, 38, 42). After 25 min of moderate exercise, chronic fatigue syndrome human patients (even with comorbid fibromyalgia), but not healthy controls, showed increased expression of ASIC3 receptors that detect muscle metabolites (34, 49). Gene expression changes might begin as soon as half an hour later and persist for 48 h after the exercise task correlating significantly with postexercise increment in severity of mental fatigue, physical fatigue, and pain (34). Female patients suffering from chronic fatigue syndrome showed a significantly decreased exercise capacity during aerobic exercises using graded increase in workload (15). Our results suggest a new inhibitory role for presynaptic ASIC1a channels, capable of finely tuned neuromuscular transmission in female mice during repetitive high-frequency stimulation. Therefore, abnormally high expression or activation of ASIC1a channels might reduce aerobic capacity of skeletal muscles by excessively inhibiting transmitter release during high-work-load, repetitive muscle exercise. Future experiments are still needed to clarify the physiological role of ASIC1a receptors during fatigue.

## GRANTS

This work was supported by grants from FONCYT-BID 1728 OC. AR. PICT 2008–2019 and FONCYT-BID 1728 OC. AR. PICT 2012–1769 (to F. J. Urbano), Wellcome Trust Grant 068941/Z/02/Z; UBACYT2011–2014 no. 20020100100427; CONICET PIP 2012–2014 11220110100768; FONCYT-BID 1728 OC. AR. PICT 2011 no. 2667 (to O. D. Uchitel), and VA Merit Review (to J. Wemmie).

## DISCLOSURES

No conflicts of interest, financial or otherwise, are declared by the author(s).

## AUTHOR CONTRIBUTIONS

Author contributions: F.J.U. and O.D.U. conception and design of research; F.J.U., N.G.L., C.M.G.-I., L.E.G., N.C., and L.G.V. performed experiments; F.J.U., N.G.L., C.M.G.-I., L.E.G., N.C., and L.G.V. analyzed data; F.J.U., L.E.G., N.C., A.W., J.W., and O.D.U. interpreted results of experiments; F.J.U. and C.M.G.-I. prepared figures; F.J.U. drafted manuscript; F.J.U., N.G.L., C.M.G.-I., L.E.G., N.C., L.G.V., A.W., J.W., and O.D.U. edited and revised manuscript; F.J.U., N.G.L., C.M.G.-I., L.E.G., N.C., L.G.V., A.W., J.W., and O.D.U. approved final version of manuscript.
